# Ontological representation, classification and data-driven computing of phenotypes

**DOI:** 10.1186/s13326-020-00230-0

**Published:** 2020-12-21

**Authors:** Alexandr Uciteli, Christoph Beger, Toralf Kirsten, Frank A. Meineke, Heinrich Herre

**Affiliations:** 1 Institute for Medical Informatics, Statistics and Epidemiology (IMISE), University of Leipzig, Leipzig, Germany; 2 SMITH Consortium of the German Medical Informatics Initiative, Leipzig, Germany; 3 Growth Network CrescNet, University of Leipzig, Leipzig, Germany; 4 Faculty of Applied Computer and Biological Sciences, University of Applied Sciences Mittweida, Mittweida, Germany; 5 LIFE Research Centre for Civilization Diseases, University of Leipzig, Leipzig, Germany

**Keywords:** Phenotype definition, Phenotype classification, Phenotype calculation, Phenotype ontology, Phenotype reasoning

## Abstract

**Background:**

The successful determination and analysis of phenotypes plays a key role in the diagnostic process, the evaluation of risk factors and the recruitment of participants for clinical and epidemiological studies. The development of computable phenotype algorithms to solve these tasks is a challenging problem, caused by various reasons. Firstly, the term ‘phenotype’ has no generally agreed definition and its meaning depends on context. Secondly, the phenotypes are most commonly specified as non-computable descriptive documents. Recent attempts have shown that ontologies are a suitable way to handle phenotypes and that they can support clinical research and decision making.

The SMITH Consortium is dedicated to rapidly establish an integrative medical informatics framework to provide physicians with the best available data and knowledge and enable innovative use of healthcare data for research and treatment optimisation. In the context of a methodological use case ‘phenotype pipeline’ (PheP), a technology to automatically generate phenotype classifications and annotations based on electronic health records (EHR) is developed. A large series of phenotype algorithms will be implemented. This implies that for each algorithm a classification scheme and its input variables have to be defined. Furthermore, a phenotype engine is required to evaluate and execute developed algorithms.

**Results:**

In this article, we present a Core Ontology of Phenotypes (COP) and the software Phenotype Manager (PhenoMan), which implements a novel ontology-based method to model, classify and compute phenotypes from already available data. Our solution includes an enhanced iterative reasoning process combining classification tasks with mathematical calculations at runtime. The ontology as well as the reasoning method were successfully evaluated with selected phenotypes including SOFA score, socio-economic status, body surface area and WHO BMI classification based on available medical data.

**Conclusions:**

We developed a novel ontology-based method to model phenotypes of living beings with the aim of automated phenotype reasoning based on available data. This new approach can be used in clinical context, e.g., for supporting the diagnostic process, evaluating risk factors, and recruiting appropriate participants for clinical and epidemiological studies.

## Background

Despite its long ago introduction in 1909 by Wilhelm Johannsen, the term ‘phenotype’ still has no generally agreed definition [1]. Usually, a phenotype is considered as an observable characteristic or trait of an organism, such as its morphology, function, behaviour or its biochemical and physiological properties [1–3]. Scheuermann et al. define a phenotype as a (combination of) bodily feature(s) (physical components, bodily qualities or bodily processes) of an organism determined by the interaction of its genetic make-up and environment [4]. From the medical perspective, clinical (clinically abnormal) and disease phenotypes (clinical phenotype characterising a single disease) are considered. According to Scheuermann et al., a disease phenotype can exist without being observed. Observed bodily features that could be of clinical relevance are called ‘Sign’( “*… observed in a physical examination and is deemed by the clinician to be of clinical significance*”) or ‘Symptom’ ( “*… observed by the patient and is hypothesized by the patient to be a realization of a disease*”) [4].

Correct determination of phenotypes plays a key role for diagnosis of diseases, evaluation of risk factors and recruitment of patients for clinical and epidemiological studies [5, 6]. One challenge is to translate phenotype algorithms, which “are most commonly represented as non-computable descriptive documents and knowledge artifacts” [7], into machine-readable form. This paper focuses on developing a general phenotype representation model that can be used for data-driven phenotype computing, i.e., software-supported determination of phenotypes based on the data of an organism. The model to be developed must support both the biological and the medical views of the phenotype notion. Recent attempts have shown that ontologies are suitable to handle phenotypes and that they can support clinical research and decision making [8–10].

There is a large ongoing initiative in Germany, the so called German Medical Informatics Initiative (MII) [11, 12] that aims at making clinical data available for research. Most German university hospitals participate in one of four funded consortia. Smart Medical Information Technology for Healthcare (SMITH) is one of these consortia [13]. Within the ongoing SMITH project, a phenotyping pipeline (PheP) will be established to systematically develop, evaluate and execute validated algorithms and models for classifying and annotating patient care data. These annotations and derivatives will be provided for triggering alerts and actions, data sharing and deep analyses of patient care and outcomes. The general design and concept of the SMITH phenotyping pipeline is presented in [14].

In this article, we propose a novel ontology-based method to model and compute phenotypes. Our approach provides an extended reasoning combining phenotypic data to derive complex phenotypes based on calculations and classifications.

## Methods

**Fig. 1:**
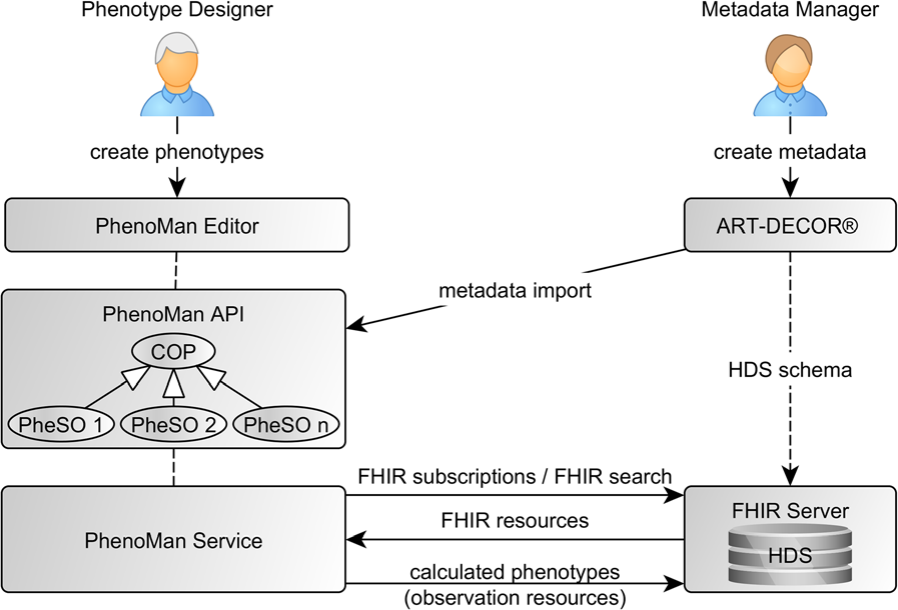
Integration of the PhenoMan. The Metadata Manager models basic data elements using ART-DECOR. The Phenotype Designer imports the ART-DECOR specification and develops phenotype models (PheSO) utilising the PhenoMan Editor. The PhenoMan requests required input data from the FHIR Server, computes phenotypes and writes the results back to the FHIR Server

Phenotypes can be derived from available data that may have been measured (quantitative data) or observed and qualitatively described (categorical data). The data can, for example, come from Electronic Health Records (EHR) (clinical data) or from a research database of a clinical/epidemiological study (research data). In SMITH, the required EHR data will be integrated into a central Health Data Storage (HDS) at each site. The integrated data is homogeneously represented in each HDS using HL7 FHIR [15] and can be queried utilising FHIR Search [16] (Fig. 1). Structured data from different source systems in hospitals as well as unstructured documents will be extracted, transformed and loaded into the HDS. Natural Language Processing (NLP) techniques are used to extract and transform relevant data from unstructured EHR documents into structured form. In SMITH and the German Medical Informatics Initiative, the software tool ART-DECOR [17] is used to specify an overarching global schema, the so-called core data set [18]. The core data subsumes the minimal set of data elements that each site (i.e., University Hospital) needs to provide in a harmonised manner. In this way, data elements are specified based on HL7 templates, their respective value sets, referenced terminologies, exemplified use scenarios and data. These specifications are the basis for the ontology-based phenotype representation in our approach.

### HL7 FHIR and FHIR Search

Healthcare records are increasingly digitised. The EHR must be discoverable and understandable. The patient data must be structured and standardised to support machine-based processing and automated clinical decision making. The FHIR (Fast Healthcare Interoperability Resources) specification is a HL7 standard for modelling and exchanging healthcare information [15]. FHIR provides a base set of resource types representing relevant clinical concepts that can be used to store and exchange data in order to solve a wide range of healthcare related problems. For each resource type, the corresponding information contents and structure are specified. The resources can be used either by themselves or combined to complex documents representing a coherent set of healthcare information [15]. The FHIR resource types include, inter alia, ‘Patient’ (“*Demographics and other administrative information about an individual or animal receiving care or other health-related services.*”), ‘Observation’ (“*Measurements and simple assertions made about a patient, device or other subject.*”) and ‘Condition’ (“*A clinical condition, problem, diagnosis, or other event, situation, issue, or clinical concept that has risen to a level of concern.*”) [15].

The FHIR Search Framework [16] is part of the HL7 FHIR standard and provides a range of operations and parameters (series of name = value pairs) to search for existing FHIR resources in the underlying repository. In the simplest case, a search is executed by performing a GET operation in the RESTful framework:

GET [base-url]/[resource-type]?name = value&...{&_format = [mime-type]}}.

e.g., GET [base-url]/Patient?gender = male.

For numeric parameter types (number, date or quantity), a value range can be defined using a prefix to the parameter value (e.g., gt = greater than, le = less or equal).

The ‘&’ (AND) operator between single search criteria is used to search for the intersection of resources that match all criteria specified by each individual search parameter (e.g., Patient?gender = male&birthdate = gt1970). To search for resources with one of the specified parameter values (OR), the values must be separated by a comma (e.g., Observation?code= http://loinc.org/3141-9, http://snomed.info/sct/27113001, i.e., weight code from LOINC or SNOMED).

The following query contains AND combinations of single criteria (code AND value-quantity) as well as OR linking of code values and can be used to search for weight observations where the weight is greater than 75 kg:

Observation?code= http://loinc.org/3141-9, http://snomed.info/sct/27113001&value-quantity=gt75//kg.

### ART-DECOR

**Fig. 2:**
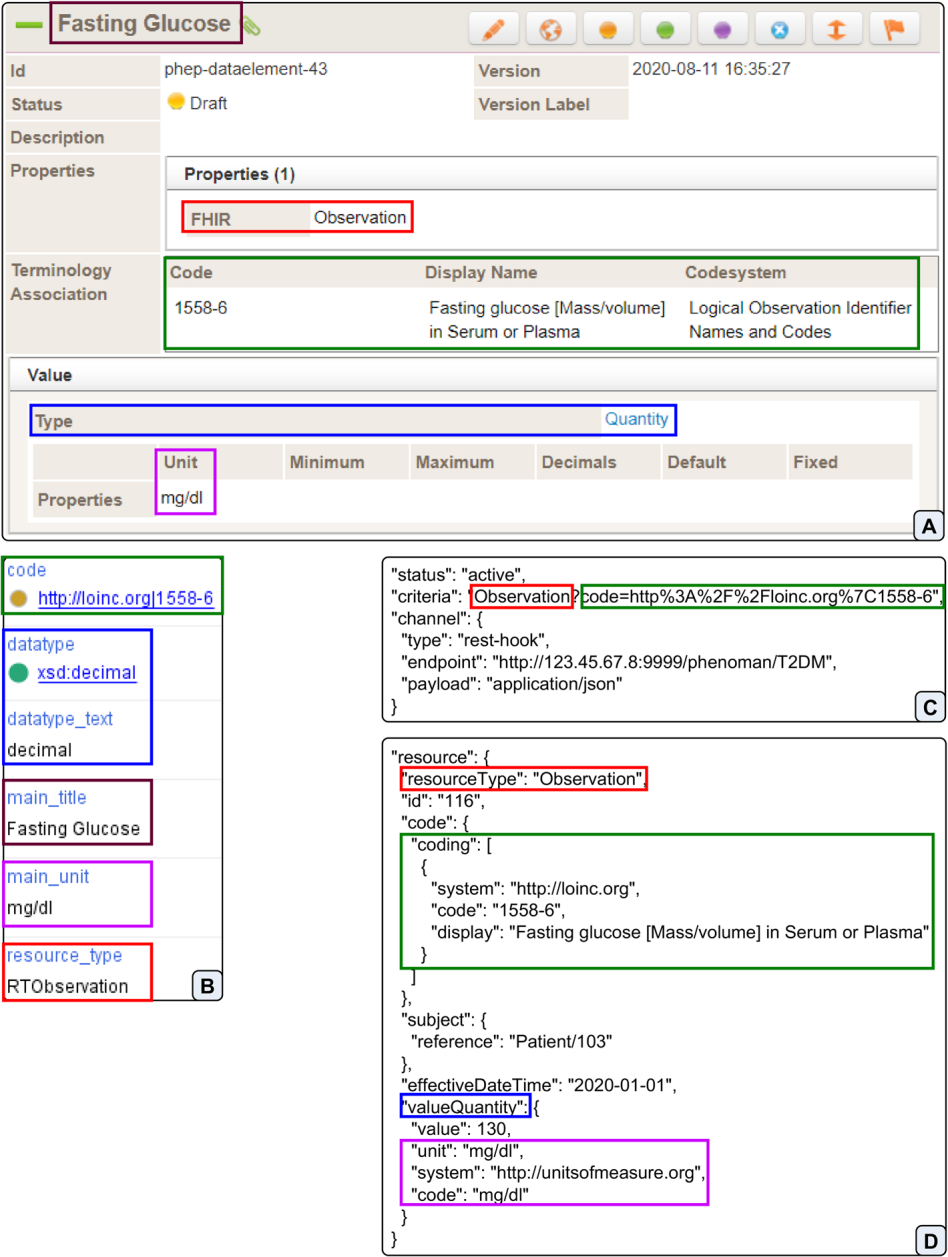
Mapping between ART-DECOR, PheSO, FHIR Subscription and FHIR Observation entities. **a** Specification of the data element ‘fasting glucose’ in ART-DECOR. **b** Annotations of the corresponding class Fasting_Glucose after importing the ART-DECOR specification into the PheSO. **c** Subscription generated for the class Fasting_Glucose. The criteria (FHIR Search query) is encoded. The original URL part is Observation?code= http://loinc.org/1558-6. **d** Observation of fasting glucose provided by FHIR Server. The observation code, value, date and the referenced patient are specified. (The same colour of the border indicates the mapping between the entities)

ART-DECOR is an open-source tool suite that supports the creation and maintenance of HL7 templates, value sets, scenarios and datasets [17]. To specify and hierarchically structure required data elements (items, concepts, variables) we use the Dataset Editor of ART-DECOR. Data elements can possess several attributes, such as name, description (in different languages) and value domain (including data type, unit and possible value set) (Fig. 2a).

One of the most important components of a data element is its terminology associations. A terminology association defines the binding of dataset concepts to relevant terminology [17]. To associate a data element with a terminology concept, the corresponding code (including the URI or ID of the terminology) must be specified. For instance, the concept ‘Fasting glucose [Mass/volume] in Serum or Plasma’ from LOINC (URI: ‘http://loinc.org’) has the code ‘1558–6’ (Fig. 2a).

Furthermore, additional properties of data elements can be defined as key-value pairs. We use this functionality to specify the mapping between the data element and the corresponding FHIR resource type (e.g., for fasting glucose, key: ‘FHIR’, value: ‘Observation’) required for phenotype computing. Depending on the resource type, different FHIR Search parameters must be used to query the relevant FHIR resources. Moreover, the different structure of the resulting resources must be considered to extract required data.

The resulting dataset specification is available in XML or JSON and can be parsed by our software.

### Ontological architecture

Our objective was to design the PhenoMan software according to the three-ontology method [19]. This method is based on interactions of three different kinds of ontologies: a task ontology (TO), a domain ontology (DO) and a top-level ontology (TLO). The TO serves as the conceptual model for the software, the DO provides the domain-specific knowledge, whereas the TLO integrates the TO and the DO and is used as foundation of them.

In our case, the Core Ontology of Phenotypes (COP, see section 'Core Ontology of Phenotypes (COP)') functions as a TO. It describes the general structure of valid phenotype specifications and thus enables the PhenoMan to create such specifications and to use them for phenotype computing. Concrete phenotype specifications (domain-specific knowledge) are represented in Phenotype Specification Ontologies (PheSO, see section 'Phenotype Specification Ontologies (PheSO)') playing the role of domain ontologies (DO) in our architecture.

For the foundation of the TO we used the General Formal Ontology (GFO) [20] as TLO. GFO has already been successfully applied for a foundation of phenotype-related notions. For instance, a novel approach to represent complex phenotypes in OWL was proposed improving the consistency and expressiveness of formal phenotype descriptions [10]. Another pillar of GFO for a grounding of phenotypes is the foundational ontology of properties, attributives and data (GFO-Data [21]) providing an extensive classification of properties (and attributives). In the current paper, we especially reference the property notion of GFO (including distinction between single and composite properties [22]) in our phenotype representation model supporting data-driven phenotype computing.

One of the advantages of the three-ontology method is that the software only needs to implement the access to entities (classes, properties) of the TO (COP), whereas the entities of the corresponding DO (PheSO) are processed dynamically. The PhenoMan uses the COP as an interface to access the PheSO entities.

Additional requirements for the ontological modelling were:

- Developing in OWL (using OWL API [23], HermiT [24] and Openllet [25])
- Modelling all attributes and relations that are relevant for reasoning as object or data properties
- Modelling all attributes and relations that are not relevant for reasoning as annotations
- Usage of general class axioms (based on ‘subclass of’) instead of equivalence classes if only one direction (‘is-a’ relation) is relevant for reasoning.

### Software design

We defined the following main requirements for the overall system (Fig. 1):

- The system must support a phenotype specification providing a GUI tool (see section 'Specification of phenotypes').
- The phenotype specifications must be saved in a standardised ontology (see sections 'Core Ontology of Phenotypes (COP)' and 'Phenotype Specification Ontologies (PheSO)').
- The system must be able to correctly compute phenotypes based on a phenotype specification (ontology) and input data (see section 'Classification and calculation of phenotypes').
- The system must support an additional implementation of mapping components for accessing required data and metadata repositories. Example components for metadata import from ART-DECOR as well as for interaction with FHIR servers (e.g., SMITH HDS) must be implemented (see sections 'Data procurement' and 'Transmission of inferred phenotype classes to the FHIR Server').

The PhenoMan accesses the FHIR Server, extracts phenotype-specific data, computes the specified phenotypes and writes the results back to the FHIR Server. For this purpose, the PhenoMan provides an API and acts as a web service (using Dropwizard [26]) (Fig. 1). The PhenoMan is implemented in Java using OWL API [23] and two reasoners, HermiT [24] and Openllet [25]. For calculations we utilize the Java Expression Evaluator (EvalEx) [27], but the integration of other libraries (e.g., for executing R scripts) or rule systems (e.g., SWIRL or Drools) is also possible. The EvalEx enables evaluating mathematical and Boolean (inter alia, Boolean operators and IF-THEN-ELSE structures) expressions and supports defining custom functions and operators.

The PhenoMan Editor^1^ is a desktop app, which is also developed with Java and bundled with the PhenoMan API. It offers a graphical user interface based on Java Swing to specify attributes of phenotype classes and categories using appropriate form fields. On saving, form content is transmitted to the PhenoMan API and written into the ontology. The editor can be executed on a local machine with a Java runtime environment 8 or higher and was developed with the aim of rapidly defining phenotype models.

### Evaluation

An evaluation of our approach was designed and conducted. The main objectives of the evaluation were to prove:

1. Correct functioning of all software components
2. Faultless communication of the software with the FHIR Server
3. Correctness of all provided phenotype specifications
4. Correct functioning of the overall system by comparison with a corresponding SPSS implementation of selected phenotypes.

We evaluated the PhenoMan at different levels. Firstly, we tested all functionalities of the PhenoMan API (especially read/write in the ontology and computing phenotypes) and the communication of the PhenoMan Service with the FHIR Server by a set of static JUnit tests using fixtures (i.e., example PheSOs and patient data).

Secondly, each phenotype specification is shipped with a structured representation (spreadsheet) of test data (input and output), such that the respective phenotype algorithm can be automatically tested. The criterion for a successful execution of the JUnit tests was a match between the results calculated by PhenoMan based on provided input data and the corresponding output data.

Finally, we selected some test case algorithms/derivatives (such as socio-economic status [28], body mass index [29], waist circumference and waist-hip ratio [30]) from the LIFE Adult study [31] running at the LIFE Research Centre for Civilization Diseases, University of Leipzig. There, derivatives are usually implemented by epidemiologists, statisticians and other researchers using the statistics software SPSS [32] and R or are database (SQL) queries and functions, which are automatically executed at night based on daily captured data. The resulting data are directly stored within the LIFE research database in tabular form. More details about participants, their invitation and consenting as well as examinations, interviews, questionnaires and taken specimen can be found in [31]. We reproduced selected SPSS derivatives using the PhenoMan and developed parameterised JUnit tests to comparatively evaluate the accuracy of the PhenoMan against the corresponding SPSS implementation at the LIFE Research Centre. The criterion for a successful comparison was a match between the results calculated by PhenoMan and SPSS software for each dataset. The performance of our approach is not a critical issue in our use case (the phenotype computing could run overnight).

This evaluation included data of thousands of LIFE participants.

## Results

### Core Ontology of Phenotypes (COP)

**Fig. 3:**
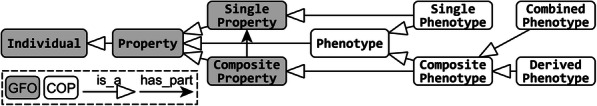
Core Ontology of Phenotypes (COP)

We developed the Core Ontology of Phenotypes (COP, Fig. 3) to model, classify and calculate phenotypes based on instance data sets (e.g., of a patient). In this article, we consider a phenotype as a dependent individual (in the sense of General Formal Ontology, GFO [20]), for example, the weight of a specific person. Hereinafter, abstract instantiable entities that are instantiated by phenotypes are called phenotype classes. For instance, the abstract property ‘weight’ possesses individual weights as instances. We distinguish between single and composite properties, and correspondingly, between single and composite phenotypes. A composite property is defined as a property that has single properties as parts [22]. Based on the definitions of single and composite properties [22], we define *single phenotypes* as single properties (e.g., age, weight, height) and *composite phenotypes* as composite properties (e.g., height and weight, BMI, SOFA score [33]) of an organism or of one of its subsystems. Properties of an organism are considered as all documentable information about it, whereby the modeller is left to decide what is relevant to the current situation. These can be, for example, observable characteristics or traits of an organism [1–3] or possible manifestations of clinical phenotypes, such as signs, symptoms or dispositions [4]. The corresponding data can be modelled using the FHIR Observation or Condition resources.

Composite phenotypes are divided into combined and derived phenotypes. A *combined phenotype* is only a combination of corresponding phenotypes (e.g., a combination of height and weight), whereas a *derived phenotype* is an additional property (e.g., BMI) derived from the corresponding phenotypes (height and weight). In the framework of GFO we modelled properties using the class gfo: Property. In the present article, composite phenotype classes are modelled using a Boolean expression based on has_part relation (e.g., weight and height: has_part some height and has_part some weight). Derived phenotype classes additionally define a calculation rule/mathematical formula (e.g., BMI = weight [kg] / height [m]^2^). Furthermore, combined phenotype classes can associate certain conditions with specific predefined values (scores), which can be used, e.g., in further formulas. For example, if bilirubin value is greater than 12 mg/dL, then the value 4 is used for the calculation of the SOFA score [33].

Additionally, we distinguish between restricted and non-restricted phenotype classes, depending on whether their extensions (set of instances) are restricted to a certain range of individual phenotypes by defined conditions or all instances are allowed. For example, the phenotype class ‘age’ is instantiated by the ages of all living beings (non-restricted), whereas the phenotype class ‘young age’ is instantiated by the ages of the young ones, e.g., if the age is below 30 years (restricted).

### Phenotype Specification Ontologies (PheSO)

We consider a phenotype algorithm as a sequence of instructions (1) to classify phenotypes (single or composite) in phenotype classes or (2) to derive additional properties (derived phenotypes) from the phenotypes of an organism. Phenotype algorithms can be implemented, for example, using a programming language or a statistics software (e.g., SPSS or R). Our approach is to separate the specification of phenotypes (models) from the implementation of corresponding algorithms. The COP provides a basic model to specify phenotypes in a standardised way, while the PhenoMan implements the general approach, common for all COP-based specifications. It is not our aim to completely model the EHR. Instead, our approach can support the modelling and calculation of selected phenotypes in a user-friendly standardised manner.

**Fig. 4:**
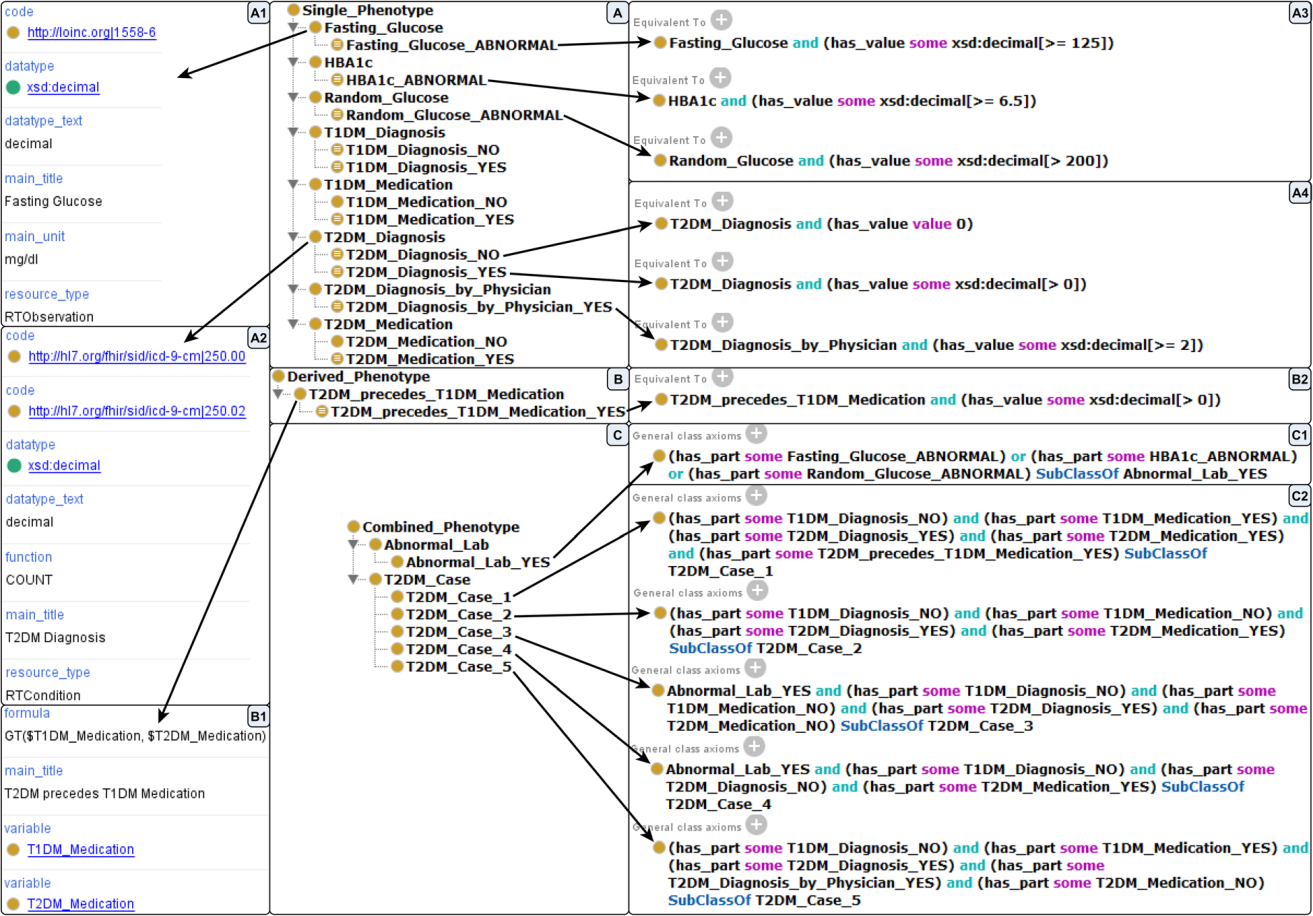
Parts of the T2DM PheSO in Protégé. Middle: Phenotype classes (**a** Single, **b** Derived, **c** Combined). Left: Example annotations of the phenotype classes. Right: Anonymous equivalent classes and general class axioms

Phenotypes are modelled in Phenotype Specification Ontologies (PheSO) using the COP. The phenotype classes and axioms (classification and calculation rules) contained in the PheSO are used by PhenoMan to execute the corresponding phenotype algorithm. PheSOs are embedded in the COP in such a way that the classes of the PheSO are subclasses of the COP classes. Every PheSO subclass of the COP classes cop: Single_Phenotype, cop: Combined_Phenotype or cop: Derived_Phenotype is a phenotype class and is instantiated by phenotypes. The direct subclasses are non-restricted (e.g., Fasting_Glucose, Fig. 4a), while the subclasses of the non-restricted phenotype classes are restricted (e.g., Fasting_Glucose_ABNORMAL, i.e., fasting glucose is greater equal 125 mg/dL, Fig. 4a3).

Phenotype classes possess various common attributes (e.g., labels, descriptions and codes of external concepts). Other attributes vary depending on the type of the phenotype class. The following are examples of such attributes:

- Non-restricted single phenotype (NSiP) class: unit of measure and optional aggregate function.
- Restricted single (RSiP) and derived phenotype (RDeP) class: restriction.
- Restricted combined phenotype (RCoP) class: Boolean expression (based on RSiP, RCoP and RDeP classes) and optional score value.
- Non-restricted derived phenotype (NDeP) class: mathematical formula and Boolean expression consisting of AND-linked variables used in the formula (NSiP and non-restricted combined phenotype (NCoP) classes). If a NCoP class is used as a variable, the RCoP classes (subclasses) of the NCoP class must have score values that should be used in the formula.

Simple attributes of the phenotype classes are defined as annotations. The logical relations between phenotype classes as well as range restrictions are represented in OWL by anonymous equivalent classes or general class axioms based on property restrictions.

### Phenotype Manager (PhenoMan)

We developed the software Phenotype Manager (PhenoMan), which implements a multistage reasoning approach combining standard reasoners (e.g., Pellet or HermiT) and mathematical calculations. This section briefly outlines the main functionality of our solution.

#### Specification of phenotypes

**Fig. 5:**
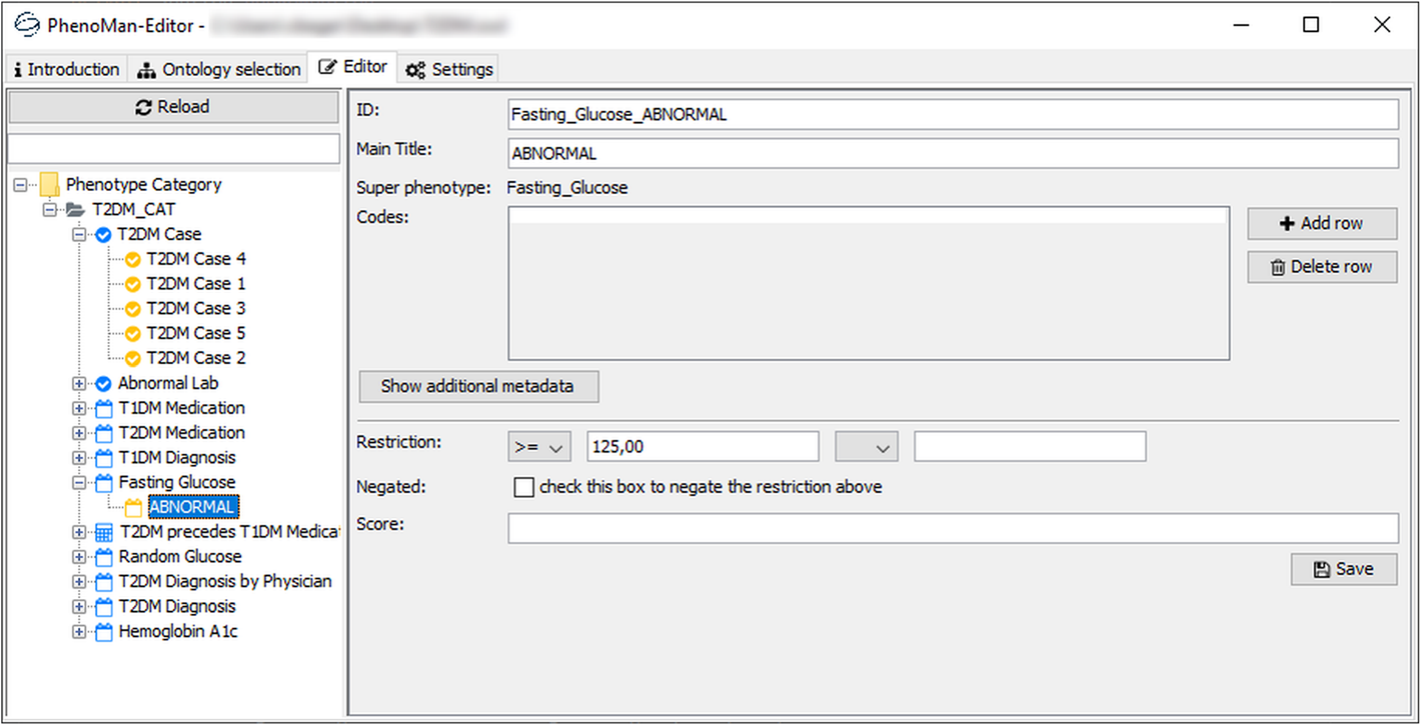
Specification of the class Fasting_Glucose_ABNORMAL with the PhenoMan Editor form. We left out some of the metadata fields for better visibility

**Fig. 6:**
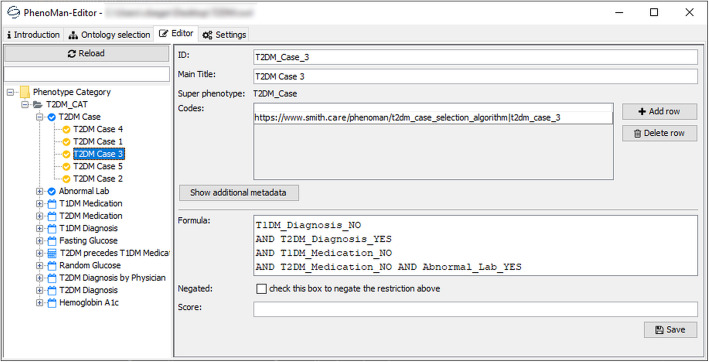
Specification of the class T2DM_Case_3 with the PhenoMan Editor form

The PhenoMan Editor is an interactive user interface for managing and developing PheSOs. The user is able to create a new PheSO or to load an existing ontology. The PhenoMan Editor provides appropriate forms to browse, create and edit categories and phenotype classes of the ontology. Value range restrictions, for example, are defined by selecting a comparison operator and entering the corresponding values (Fig. 5). Boolean expressions are built by drag-and-dropping the phenotype classes from the left side into the expression form field and entering relevant operators (Fig. 6). After submission, the form data is transmitted to the PhenoMan API and is stored in the actual PheSO.

Furthermore, an ART-DECOR specification (XML) of relevant data elements can be imported in the PheSO. For each data element, a NSiP class is generated. All relevant attributes (name, codes, FHIR resource type, data type, unit, etc.) specified in ART-DECOR are defined as annotations of corresponding classes (Fig. 2a, b).

#### Data procurement

After starting the PhenoMan Service, FHIR subscriptions (rest-hooks) [34] are generated and transmitted to the FHIR Server. The structure of the subscription resource is very simple. The main parts of the resource are the criteria and the channel. The FHIR Server uses the criteria (FHIR Search query) to determine resources for which notifications have to be generated. When resources are identified (after creating or updating) meeting the criteria, a notification is sent to the address (‘endpoint’) specified in the section ‘channel’.

In the configuration file of the PhenoMan, a directory containing all available phenotype specifications (PheSOs) as well as the address (URL) of the PhenoMan service (including the PheSO name) are defined. For each NSiP class of each available PheSO (in the defined directory) a subscription is created. To generate the subscription criteria (FHIR Search query), the PhenoMan uses the resource type and codes specified in the corresponding NSiP class as annotations (Fig. 2b, c). The ‘endpoint’ attribute is automatically filled with the URL of the PhenoMan service defined in the configuration file. The remaining parts of the subscription resource (‘status’, ‘type’ and ‘payload’) take default values (‘active’, ‘rest-hook’ and ‘application/json’) (Fig. 2c).

After receiving a notification (including the complete resource, Fig. 2d), the PhenoMan Service requests further resources (for all other NSiP classes of the corresponding PheSO) using FHIR Search. The generated FHIR Search queries are primarily based upon the codes specified for the NSiP classes (similarly to subscription criteria), contain a reference to the patient and can additionally support possible aggregate functions.

#### Classification and calculation of phenotypes

After receiving required resources, the PhenoMan starts inferring phenotypes.

First, the relevant information is extracted from received resources and inserted into the ontology. On the one hand, the individual properties (single phenotypes) are inserted as instances of the direct subclasses of cop: Single_Phenotype and the values are modelled as property assertions based on the has_value relation. On the other hand, a composite phenotype is defined as an instance of the class cop: Composite_Phenotype, which combines all the single phenotype instances using property assertions based on has_part relation. Then, our multistage reasoning algorithm is executed. The algorithm consists of the following steps:

1. *Classification step*. A standard reasoner classifies the existing instances (assignment to classes).
  - Single phenotype instances are classified in RSiP classes based on property restrictions.
  - The composite phenotype instance is classified in RCoP classes based on the specified Boolean expression and inferred RSiP, RCoP and RDeP classes.
  - The composite phenotype instance is classified in NDeP classes based on the specified Boolean expression and corresponding NSiP and NCoP classes. In this case, all variable values required for calculating formulas are present.
  - Available instances of NDeP classes (representing calculated values) are classified in RDeP classes based on property restrictions.
2. If no new NDeP classes get an instance, the execution of the algorithm stops. All inferred phenotypes (inferred classes including metadata and calculated values) are returned.
3. *Calculation step*. The formula of each NDeP class having an instance is calculated based on variable values (values of the corresponding single phenotype instances or scores of the inferred RCoP classes). A new instance of the NDeP class representing the calculated value is created and associated with the composite phenotype instance (using has_part).
  The algorithm goes back to step 1.

In the case of complex phenotypes, the classification and calculation steps can be executed several times (in a loop). That is the case if a NDeP class has subclasses, i.e., RDeP classes, which are in turn used in combined phenotypes. Both steps are repeated until all formulas are calculated and all phenotypes are classified.

The PhenoMan supports 4 primitive data types xsd:decimal, xsd:string, xsd:boolean and xsd:date. All other complex data types (e.g., FHIR code or quantity) are mapped to the primitive data types (e.g., code to xsd:string and quantity to xsd:decimal with additional unit attribute, Fig. 2a, b). Furthermore, the PhenoMan provides, inter alia, aggregate functions, Boolean, date and measurement unit arithmetic, integration of external terminologies as well as reading and writing FHIR resources.

#### Transmission of inferred phenotype classes to the FHIR Server

**Fig. 7:**
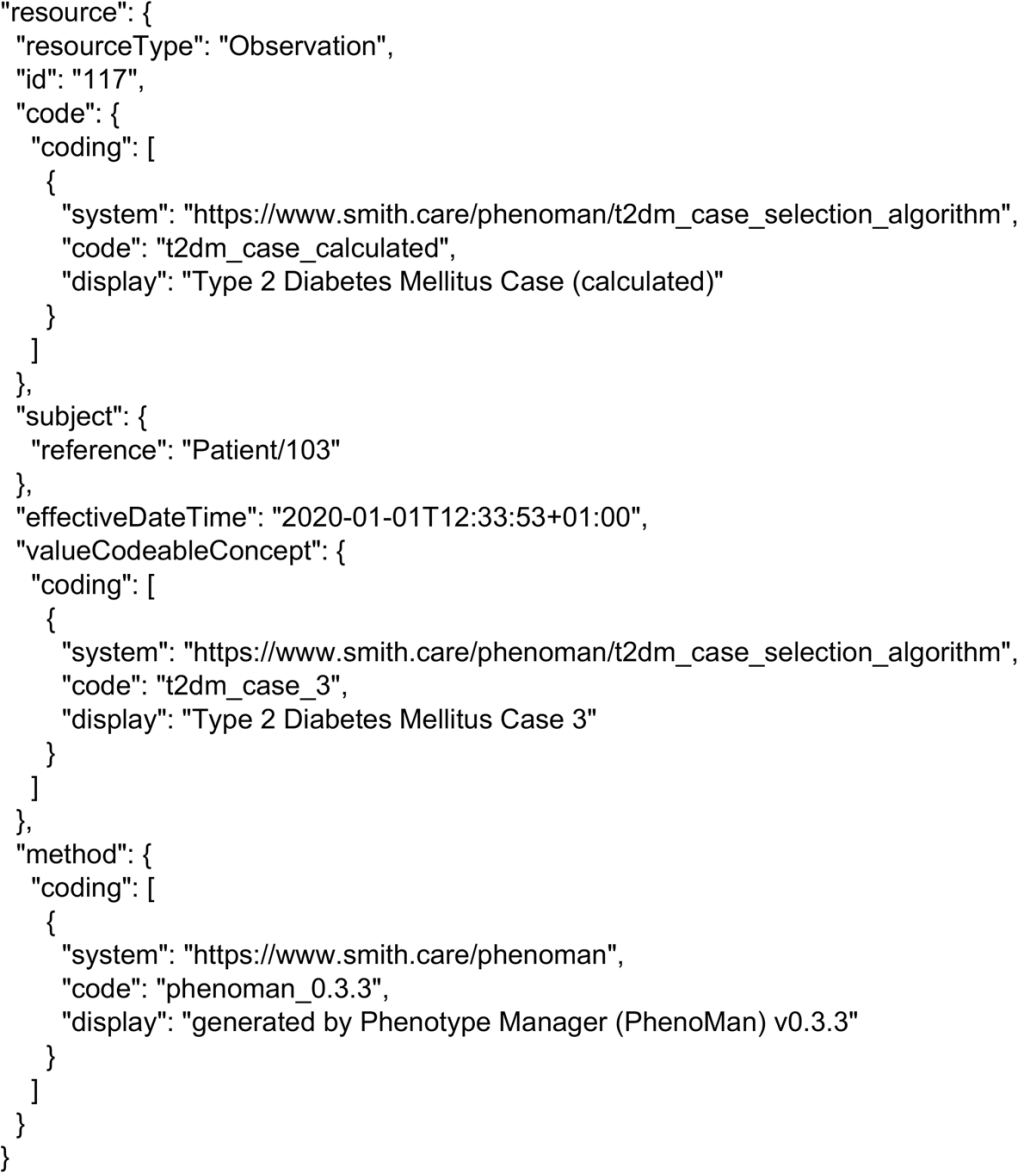
T2DM case 3 Observation. For the Patient/103, an Observation resource was generated including the code (t2dm_case_calculated), the value (t2dm_case_3) and the observation method (generated by PhenoMan)

PhenoMan returns all inferred combined and derived phenotype classes as Observation resources and transmits them to the FHIR Server. The generated resources can have a numeric or a code data type. Numeric observations are used for storing numeric values (i.e., calculated values of derived phenotypes or score values of combined phenotypes), whereas the code observations are intended for concepts of a terminology or a value set. The specified codes of the non-restricted phenotype classes are utilised in the resulting resources to complete the ‘code’ attribute. The calculated values, scores or codes of the inferred restricted phenotype classes are used in ‘valueQuantity’ or ‘valueCodeableConcept’ attributes (Fig. 7).

#### Export capabilities

**Fig. 8:**
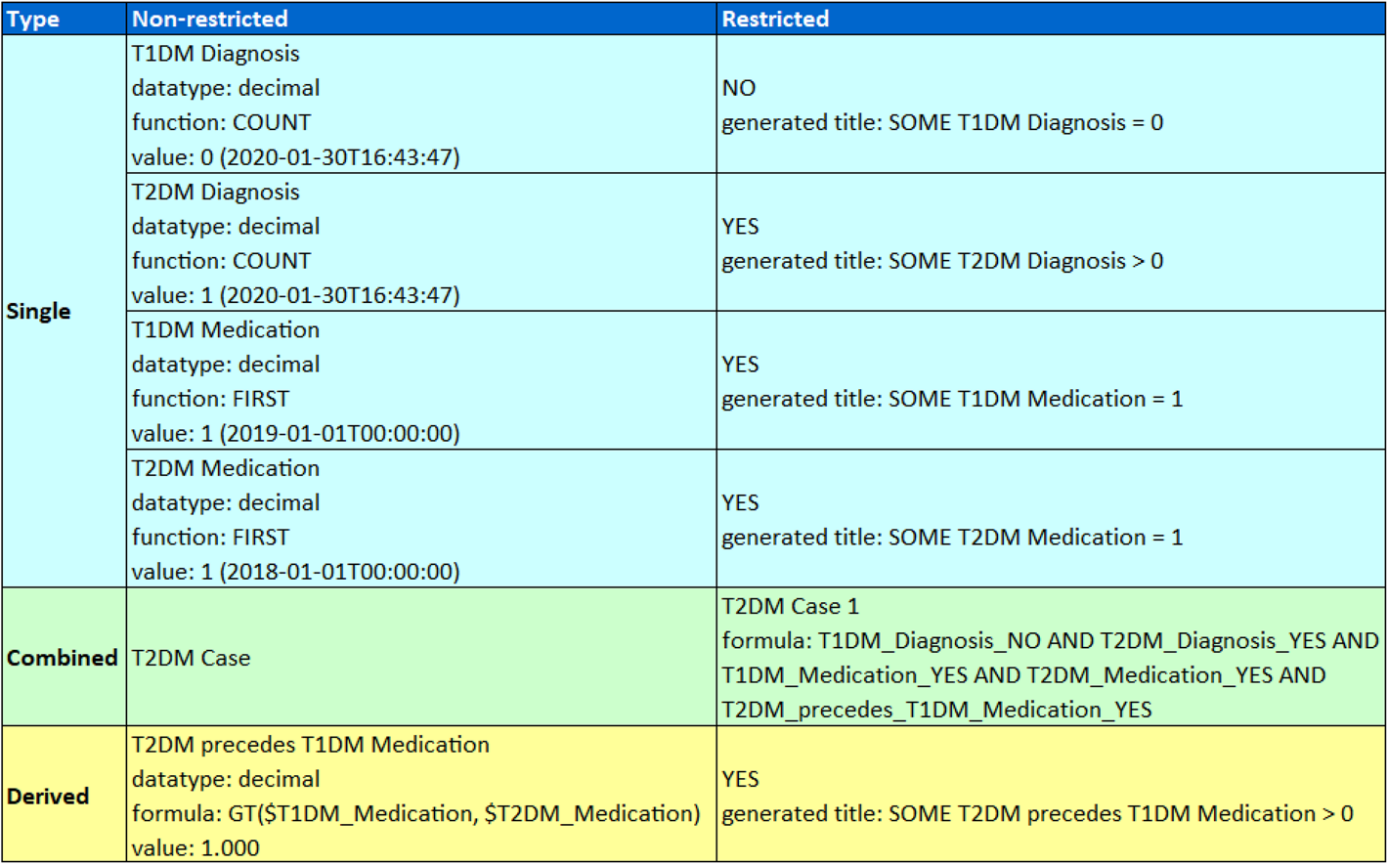
T2DM case 1 reasoner report. Left: Phenotype class types from the COP (single, combined, derived). Middle: Non-restricted classes of the PheSO (the NSiP classes contain the current input value, the NDeP classes the calculated value). Right: Restricted classes inferred by PhenoMan based on the input data

The PhenoMan can export the complete PheSO or generate reasoner reports (structured descriptions of all inferred phenotypes) in Excel format. A tabular reasoner report contains three columns: ‘Type’, ‘Non-restricted’ and ‘Restricted’. In the first column the type (single, combined or derived) of the inferred phenotype is presented. In the next two columns, the specified or derived information about the resulting phenotypes (restricted and non-restricted) is displayed (Fig. 8).

**Fig. 9:**
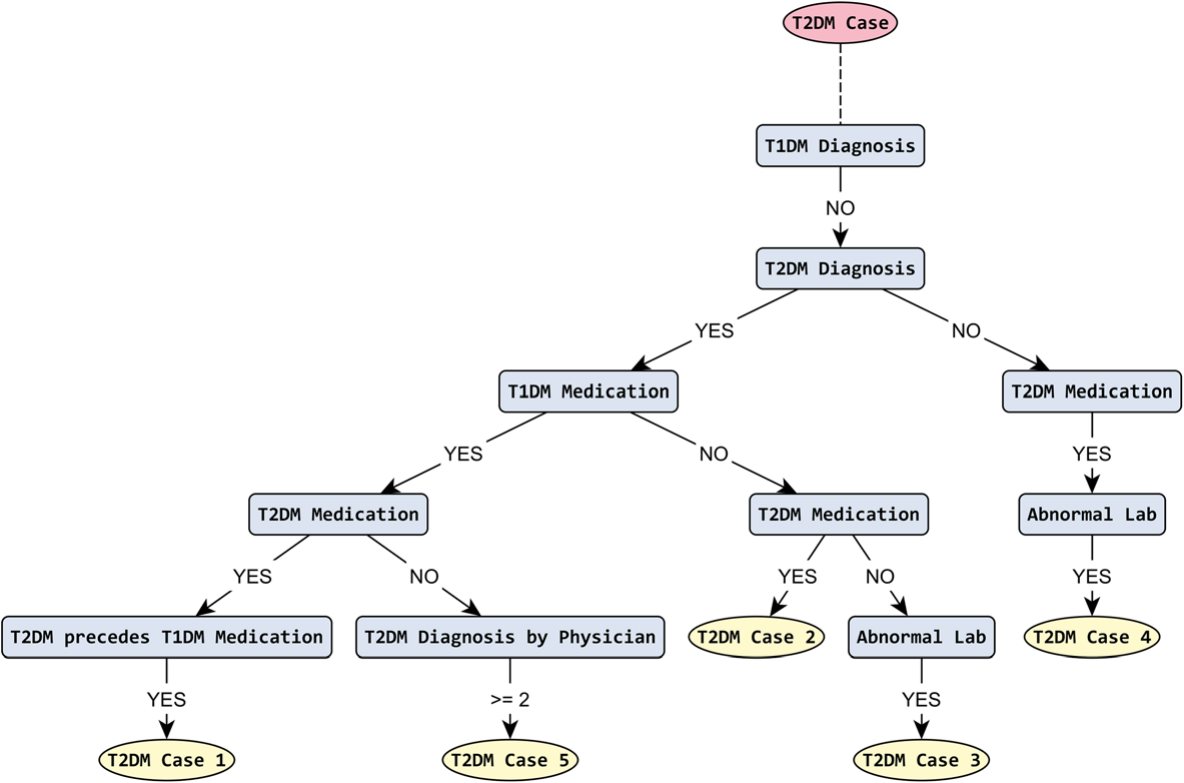
T2DM decision tree. The tree was generated by PhenoMan in GraphML format and was processed using an automatic layout [35] of the yEd Graph Editor [36]. Nonetheless, it is also possible to generate the complete tree as image in PNG format

Additionally, the PhenoMan can generate the graphical representation of combined phenotype classes in the form of decision tree (or flowchart) diagrams. The PhenoMan translates the Boolean expressions of every RCoP class into disjunctive normal form and considers each conjunction as a possible path of the tree. Then, the paths are grouped on shared nodes and form a tree structure (Fig. 9).

### Example

We illustrate our approach by means of an example algorithm for determining Type 2 Diabetes Mellitus (T2DM) cases presented by PheKB.org [37]. The T2DM algorithm requires the following data elements to be extracted from the EHR:

- Counts of T1DM and T2DM diagnoses (identified by ICD-9 billing codes),
- The earliest dates of T1DM and T2DM medications (identified by RxNorm codes),
- Laboratory values (fasting blood glucose, random blood glucose and hemoglobin A1c, identified by LOINC codes) as well as
- Physician-entered diagnoses (derived from encounter or problem list sources only).

#### Modelling of single data elements using ART-DECOR

As a first step, required data elements (items, concepts, variables, single phenotype classes) representing single patient characteristics relevant for determining the T2DM (T1DM and T2DM diagnoses, T1DM and T2DM medications, fasting blood glucose, random blood glucose, hemoglobin A1c as well as physician-entered diagnoses) must be modelled using ART-DECOR. Labels, descriptions, codes from external terminologies, data types, units, etc. are specified. Additionally, every data element must be associated (as property) with a FHIR resource type in order to ensure the correct search and extraction of the instance values from the respective resource. Laboratory values are represented in FHIR as Observations, while diagnoses are usually specified using the Condition resource. The Fig. 2a shows the filled form of fasting glucose in ART-DECOR.

The resulting specification is provided by ART_DECOR as a XML or JSON file.

#### Modelling of phenotypes using PhenoMan Editor

The user imports the ART-DECOR specification in the ontology (PheSO) utilizing the PhenoMan Editor. For each data element, a NSiP class (Fasting_Glucose, HBA1c, Random_Glucose, T1DM_Diagnosis, T2DM_Diagnosis, T1DM_Medication, T2DM_Medication and T2DM_Diagnosis_by_Physician) including relevant annotations is generated (Fig. 4a, a1, a2).

Furthermore, aggregate functions (e.g., COUNT, FIRST, LAST, MIN, MAX) can be defined for NSiP classes. For instance, the T2DM algorithm does not require to process the complete data of all diagnosis resources, it is sufficient to count the resources. Therefore, the function COUNT is defined for the class T2DM_Diagnosis (Fig. 4a2). The both medication classes (T1DM_Medication and T2DM_Medication) are associated with the function FIRST, because only the earliest date of medications is relevant for the algorithm.

Next, the RSiP classes (Fasting_Glucose_ABNORMAL, Random_Glucose_ABNORMAL and HBA1c_ABNORMAL) for value range restrictions are defined as subclasses of the NSiP classes (Fig. 4a, a3, Fig. 5). For every RSiP class, the anonymous equivalent class is created that represents the corresponding restriction. The restrictions of other NSiP classes (e.g., T2DM_Diagnosis or T2DM_Diagnosis_by_Physician) are based on the counts of the corresponding resources (e.g., T2DM_Diagnosis_NO: if the count of T2DM_Diagnosis = 0 or T2DM_Diagnosis_by_Physician_YES: if the count of T2DM_Diagnosis_by_Physician > = 2, Fig. 4a4).

Mathematical calculations are modelled using NDeP classes. The formula ‘GT($T1DM_Medication, $T2DM_Medication)’ (‘GT’ stands for Greater Than) defined for the class T2DM_precedes_T1DM_Medication expresses a comparison of the T1DM and T2DM medication dates (Fig. 4b, b1). The dollar sign in the variable name indicates that not the medication value itself but the entry date of the medication is used in the formula. The formula returns − 1, 0 or 1 depending on whether the first operand is less than, equal to or greater than the second operand. The value − 1 is also returned if one of the operands is missing. The RDeP class T2DM_precedes_T1DM_Medication_YES and the corresponding restriction are specified similarly to RSiP classes (Fig. 4b, b2).

The next step is to model the abnormal lab, i.e., if either random glucose or fasting glucose or HBA1c is abnormal. For this purpose, we define the non-restricted combined phenotype (NCoP) class Abnormal_Lab and the RCoP class Abnormal_Lab_YES with the corresponding Boolean restriction (disjunction) formalised as general class axiom (Fig. 4c, c1).

Finally, the T2DM case selection rules are modelled using the NCoP class T2DM_Case as well as the five RCoP classes (one for each case type) including Boolean restrictions (Fig. 4c, c2, Fig. 6). For instance, case 1 occurs when the T1DM diagnosis is missing but a T2DM diagnosis as well as both medications (T1DM and T2DM) are present and the first T2DM medication precedes the first T1DM medication. The abnormal lab, the presence of a T2DM diagnosis and the absence of T1DM diagnosis and both medications indicate the case 3.

The resulting ontology and additional material (graphical and tabular representation, reasoner reports) are publicly available [38].

#### Execution of the PhenoMan Service

The PheSO (OWL file) created by the PhenoMan Editor is saved in the directory specified in the configuration file. After starting the PhenoMan Service, subscriptions for each NSiP class of the T2DM PheSO are created. The subscription for the NSiP class Fasting_Glucose, for example, is intended to identify Observation resources with the LOINC code ‘1558–6’ (criteria) (Fig. 2c). After receiving a fasting glucose resource, other required resources are queried. The query for T2DM diagnosis, for instance, includes the additional FHIR Search parameter _summary = count to express the aggregate function COUNT:

Condition?code=http://hl7.org/fhir/sid/icd-9-cm/250.00, http://hl7.org/fhir/sid/icd-9-cm/250.02&subject=Patient/103&_summary=count.

In this case, the server returns a bundle with only the number of resources matching the query. To realise the function FIRST, the combination of _sort (sorting by date) and _count (_count = 1) is used. The following T2DM medication query returns only the first resource matching the criteria:

MedicationStatement?code=http://www.nlm.nih.gov/research/umls/rxnorm/25789, http://www.nlm.nih.gov/research/umls/rxnorm/10633&subject=Patient/103&_sort=effective&_count=1

(Some codes were omitted in both queries).

As soon as all required FHIR resources are present, the PhenoMan starts the phenotype computing. Suppose, the input resource set consists of only a fasting glucose (Fig. 2d) and a T2DM diagnosis resource. In this case, the single phenotype instances of the classes Fasting_Glucose and T2DM_Diagnosis are created and the values are modelled as property assertions based on the has_value relation (e.g., ‘has_value 130’ for Fasting_Glucose and ‘has_value 1’ for T2DM_Diagnosis (due to the COUNT function)). Then, a composite phenotype instance is defined, which combines both single phenotype instances using property assertions based on has_part relation. In the first step (classification step), a standard reasoner classifies the single phenotype instances in restricted classes. In our example, the instance of Fasting_Glucose is classified in the class Fasting_Glucose_ABNORMAL (i.e., the fasting glucose value is > = 125 mg/dL, Fig. 4a, a3) and the instance of T2DM_Diagnosis in the class T2DM_Diagnosis_YES (because the count of the T2DM diagnoses is > 0, Fig. 4a, a4). Next, the composite phenotype instance is classified in the RCoP classes Abnormal_Lab_YES (because the fasting glucose is abnormal, Fig. 4c, c1) and T2DM_Case_3 (because all conditions of the corresponding Boolean expression are fulfilled, Fig. 4c, c2). In this case, further phenotypes can not be derived or calculated.

Let us consider another example. Suppose, the input data set contains a T2DM diagnosis, a T1DM and a T2DM medication (T2DM precedes T1DM medication). The classification step is similar to the first example. The corresponding single phenotype instances are classified in the classes T1DM_Diagnosis_NO, T2DM_Diagnosis_YES, T1DM_Medication_YES and T2DM_Medication_YES. In the next step (calculation step), the formula of the NDeP class T2DM_precedes_T1DM_Medication (Fig. 4b, b1) can be calculated by PhenoMan. It inserts the variable values (the dates of the both medications) in the formula and starts the calculation. After the calculation step, the classification step must be performed again. The calculated instance of T2DM_precedes_T1DM_Medication is classified in the class T2DM_precedes_T1DM_Medication_YES (because the formula returns 1, Fig. 4b, b2). Then, the composite phenotype instance is classified in the RCoP class T2DM_Case_1 (Fig. 4c, c2) and the PhenoMan finishes the calculation.

Finally, the PhenoMan generates the Observation resource for the resulting T2DM case and transmits it to the FHIR Server. In our example, we encode the possible T2DM cases using a code system (https://www.smith.care/phenoman/t2dm_case_selection_algorithm), one code identifying the observation (t2dm_case_calculated) and five codes for possible values (t2dm_case_1, t2dm_case_2, t2dm_case_3, t2dm_case_4 and t2dm_case_5). The resulting Observation resource for case 3 is illustrated in Fig. 7.

Additionally, we can generate a tabular reasoner report or a decision tree diagram.

An example reasoner report for case 1 is shown in Fig. 8.

The class T2DM_Case and its subclasses (T2DM_Case_1, T2DM_Case_2, etc.) are very suitable for the representation as a decision tree. The decision tree generated by PhenoMan (Fig. 9) looks similar to the flowchart specified by PheKB.org [37].

### Evaluation results

The evaluation has demonstrated that all components of our solution function correctly. All JUnit tests were successful and showed no difference between specified and calculated results. The comparison between the PhenoMan and the SPSS calculation has also succeeded. Although the performance of our approach is not a critical issue in our use case, we measured the execution time of the PhenoMan. The calculation of the socio-economic status (complex algorithm requiring multiple reasoner runs), for example, takes approximately 0.5 s per dataset. This performance is completely sufficient for our use case.

In summary, PhenoMan correctly computes phenotypes based on valid phenotype specifications (PheSO) and input data. Additionally, validating phenotype specifications (PheSOs) before deploying them in a productive environment is an extremely useful feature of the PhenoMan.

## Related work

We developed a novel approach to support ontological modelling and reasoning of phenotypes. In contrast to [8, 9], our solution serves to determine and to classify phenotypes based on instance data (e.g., EHR). Moreover, the proposed reasoning process includes calculation of mathematical formulas at runtime.

Very similar to our approach, Fernández-Breis et al. [39] propose to take advantage of the best features of EHR standards and ontologies. The authors developed methods allowing a direct use of EHR data for the identification of patient cohorts leveraging current EHR standards and semantic web technologies. In [39], openEHR [40] archetypes were used as EHR standard. An ontological infrastructure was designed including different ontologies for representing domain entities (colorectal-domain), the rules for determining the risk level and the data. The mappings between the phenotyping archetype and the colorectal-domain ontology were defined and are automatically executed on the archetyped data instances to generate the OWL dataset. The data is then transformed into OWL, where the classification is performed.

Papež et al. [41] evaluated OWL/RDF for enabling computable representations of EHR-driven phenotyping algorithms. A proof-of-concept application using the OWL API and the HermiT reasoner was developed. The phenotype algorithms are specified based on a rudimentary core ontology without the possibility to model and execute mathematical calculations. The authors utilised a simple diabetes phenotype algorithm as an example and validated their approach against a selected set of desiderata proposed by Mo et al. [7]. The evaluation showed that OWL/RDF is potentially sufficient to store phenotyping algorithms, as it is versatile enough to meet most of the desiderata.

We use HL7 FHIR as a standard for exchanging healthcare information in the SMITH infrastructure. But the main difference to the both ontological approaches described above lies in our three-level ontological architecture. The COP is founded by GFO and provides a framework for developing PheSOs. In this way, each particular phenotype model specified as a PheSO has the same standardised structure and can be executed by PhenoMan in the same manner. A further advantage of our solution is that the PhenoMan supports classification as well as calculation tasks and works directly with FHIR format, so that no further transformations are required. The mapping between EHR data and ontology is performed by PhenoMan automatically using terminology associations, which are defined for each data element in ART-DECOR (and imported into ontology) as well as in FHIR resources (e.g., Observation).

The main objective of SHARPn [42] is to develop methods and modular open-source resources for enabling secondary use of EHR data for high-throughput phenotyping. The phenotype algorithms are specified based on Quality Data Model (QDM) [43] and represented in the HL7 Health Quality Measures Format (HQMF or eMeasure) [44]. According to the authors, there are two main challenges. Firstly, data elements in an EHR may not be represented in a format consistent with the QDM. Secondly, an EHR typically does not natively have the capability to automatically consume and execute eMeasure logic. To address these challenges, a translator tool was developed that converts QDM-defined phenotyping algorithm criteria into executable Drools rules scripts.

The Phenotype Execution and Modeling Architecture (PhEMA) [45] is an open-source infrastructure for standards-based authoring, sharing, and execution of phenotyping algorithms. Similarly to SHARPn, PhEMA uses QDM and HQMF to model phenotype definitions. Phenotyping algorithms are represented using the PhEMA Authoring Tool (PhAT), are exported from the PhAT into executable KNIME [46] workflows and are executed against data warehouses or data repositories.

In contrast to the rule- or workflow-based description of phenotyping algorithms, we use an ontology-based one. Our approach is rather generic and enables a standardised and structured modelling as well as the reuse of phenotyping algorithms and their parts (e.g., concepts and restrictions). Furthermore, the PhenoMan is compatible with the native representation of EHR data (HL7 FHIR) in the SMITH infrastructure and does not need an additional import of the data into a data warehouse.

In [47] a FHIR-compatible model was designed to support capture of cancer clinical data. Our approach allows the modelling of different phenotypes based on a core ontology (COP) and is independent of the EHR representation standards. The interpretation of FHIR data and the mapping to specified phenotypes using terminology associations are provided by PhenoMan.

A method to enable automated transformation of clinical data into OWL ontologies is presented in [48]. The developed system generates OWL representations of openEHR archetypes and automatically transforms openEHR data to OWL individuals. In our approach, the phenotypes are directly modelled in the ontology and are automatically mapped to the EHR data. Moreover, our solution supports classification as well as calculation of phenotypes.

As described in section ‘Phenotype Specification Ontologies’, phenotypes can possess links to concepts of external ontologies. For instance, they may be annotated with concepts of anatomic structures (e.g., Foundational Model of Anatomy [49]), or situations, respective processes, where phenotypes are observed (e.g., electrocardiographic monitoring). The linkage is similar to the Entity-Quality method [50] (entity: anatomic structure or process, quality: phenotype) and may improve comparison of COP across multiple domains.

Hoehndorf et al. [9] proposed the PhenomeNET for incorporation of phenotype ontologies from different species. PhenomeNET can predict orthologous genes with common pathways and common related diseases. Apart from the different interpretation of the term ‘phenotype’, the main focus of our attempt is to deduce complex phenotypes from a set of basic phenotypes of an individual.

The Human Phenotype Ontology (HPO) [8] associates phenotypic abnormalities with underlying diseases and participating genes, whereas COP can contain all sorts of properties of an organism (including non-abnormalities). Currently, COP does not offer weights for phenotype-disease relations, like HPO does to sort diseases for a phenotype set by relevance. We will investigate ways to add this functionality to COP in future.

## Discussion

Mo et al. [7] propose 10 desired characteristics for a flexible, computable phenotype representation model. In this section, we discuss our implementation of the proposed desiderata.

### Structure clinical data into queryable forms

In the SMITH project, the patient data is structured and integrated in a Health Data Storage (HDS) according to HL7 FHIR. FHIR Search is used as a query language. PhenoMan supports querying and writing FHIR resources to populate the PheSO respectively to write the inferred phenotypes back to the server. Additionally, we investigate the future application scenarios of more expressive query languages, such as FHIRPath [51] and Clinical Quality Language (CQL) [52].

### Recommend a common data model, but also support customization for the variability and availability of EHR data among sites

HL7 FHIR is used as a common EHR data model. The FHIR specification supports adaptation to particular contexts of use by means of profiling [53].

### Support both human-readable and computable phenotype representations

The PheSOs are computable representations of phenotype models. They are implemented in OWL and can be edited using the PhenoMan Editor or common ontology editors such as Protégé. The PhenoMan interprets PheSOs based on the COP and infers specified phenotypes. Additionally, the PhenoMan provides human-readable representations in graphic (diagrams) or tabular (spreadsheets) form.

### Implement set operations and relational algebra

The ontological representation supports intersection, union and negation operations as well as existential and universal quantifiers in accordance with OWL DL. To extend the expression possibilities, First-Order Logic can be considered in the future work.

### Represent phenotype criteria with structured rules

Our multistage reasoning approach supports structured specification of phenotype models from single phenotypes (non-restricted and restricted) through combined phenotypes (Boolean expressions) to derived phenotypes (mathematical calculations). The PhenoMan iteratively processes the individual steps (classification and calculation rules) in the proper order until all specified phenotypes are inferred. It supports Boolean operations (conjunction, disjunction and negation), specification of value ranges (as intervals or enumerations) and aggregate functions (for multiple values of single phenotypes as well as for values calculated based on different datasets).

### Support defining temporal relations between events

All FHIR resource types processed by PhenoMan possess one or more date attributes. The relevant date attributes were implemented and associated with the corresponding resource types. When the PhenoMan receives resources from the FHIR Server, the dates of the resources are considered and can be used as variable values in formulas of NDeP classes (the dollar sign in the variable name indicates the resource date as variable value, Fig. 4b1).

### Use standardised terminologies, ontologies, and facilitate reuse of value sets

In the SMITH project, the metadata (catalogue of items, data elements) is specified using the software ART-DECOR [17]. ART-DECOR is very suitable to specify terminology associations (e.g., LOINC, SNOMED, ICD, RxNorm) and value sets. The PhenoMan integrates the metadata from ART-DECOR and uses the specified terminology associations and value sets (codes) to query the HDS and to write the inferred phenotypes.

### Define representations for text searching and natural language processing

In SMITH, a NLP module is upstream that extracts and transforms relevant data from unstructured EHR documents into structured form. The structured data is then integrated in the HDS, so that the PhenoMan can work only with structured data.

### Provide interfaces for external software algorithms

We use the Java Expression Evaluator (EvalEx) [27] for mathematical calculations, but the integration of other libraries (e.g., for executing R scripts) or rule systems (e.g., SWIRL or Drools) is also possible and will be evaluated in future work.

### Maintain backward compatibility

Our ontological phenotype representation model is robust to changes in EHR data, used terminologies and standards. If, for instance, in our T2DM example both ICD9 and ICD10 must be supported, the missing codes (ICD10) have to be completed in the corresponding PheSO. Additionally, we develop a component to specify and automatically integrate required code mappings into the ontology. The changes in the EHR data model have no impact on the phenotype specifications (PheSOs). The PhenoMan contains a FHIR mapping module implementing all required functionalities to extract relevant information from FHIR resources. Supported resource types are offered for selection during the phenotype specification and are saved in the corresponding PheSO. If the EHR data model changes, it is only necessary to re-implement the mapping module. The switch from FHIR r3 to r4 has already been done.

## Conclusion

We developed a novel ontology-based method to model phenotypes of living beings with the aim of automated phenotype reasoning based on instance data (e.g., patient data from EHR). Our solution includes an enhanced iterative reasoning process combining classification tasks with mathematical calculations at runtime. This new approach can be used in clinical context, e.g., for supporting the diagnostic process, evaluating risk factors or recruiting appropriate participants for clinical or epidemiological studies. About 20 phenotype models have already been specified and the ontology as well as the reasoning method were successfully evaluated.

Our approach has currently the following limitations: 1. only structured data can be considered (a NLP module is upstream); 2. mathematical calculations are only possible for individual data sets (no statistical evaluations over multiple data sets).

An integration of more complex algorithms into the reasoning process is possible and has to be investigated in respect of accessing external libraries (e.g., R scripts). The current formalism will be extended in the future to optimise the implementation of selected desiderata expounded by Mo et al. [7].

## Data Availability

The PhenoMan Editor, developed ontologies and generated phenotype representations (diagrams, spreadsheets) [38] used in this paper are publicly available. Further software components are available from the corresponding author on reasonable request. At the end of the SMITH project, the consortium will take a final decision about licensing and usage of developed software components.

## Abbreviations

COP: Core Ontology of Phenotypes
PheSO: Phenotype Specification Ontology
PhenoMan: Phenotype Manager
NSiP class: Non-restricted single phenotype class
RSiP class: Restricted single phenotype class
NCoP class: Non-restricted combined phenotype class
RCoP class: Restricted combined phenotype class
NDeP class: Non-restricted derived phenotype class
RDeP class: Restricted derived phenotype class
TO: Task ontology
DO: Domain ontology
TLO: Top-level ontology

## Footnotes

1 Source code and releases of the PhenoMan Editor are available on GitHub under the GPL-3.0 license: https://github.com/Onto-Med/PhenoMan-Editor
